# Simple Coumarins from *Peucedanum luxurians* Fruits: Evaluation of Anxiolytic Activity and Influence on Gene Expression Related to Anxiety in Zebrafish Model

**DOI:** 10.3390/ijms24108693

**Published:** 2023-05-12

**Authors:** Jarosław Widelski, Natalia Kasica, Monika Maciąg, Simon Vlad Luca, Barbara Budzyńska, Dafina Fondai, Piotr Podlasz, Krystyna Skalicka-Woźniak

**Affiliations:** 1 Medicinal Plant Unit, Department of Pharmacognosy, Medical University of Lublin, 20-093 Lublin, Poland; 2 Department of Animal Anatomy, Faculty of Veterinary Medicine, University of Warmia and Mazury in Olsztyn, 10-719 Olsztyn, Poland; natalia.kasica@uwm.edu.pl; 3 Independent Laboratory of Behavioral Studies, Medical University of Lublin, 20-093 Lublin, Poland; monika.maciag@umlub.pl (M.M.); barbara.budzynska@umlub.pl (B.B.); 4 Biothermodynamics, TUM School of Life Sciences, Technical University of Munich, 85354 Freising, Germany; vlad.luca@tum.de; 5 Department of Pharmacognosy, Grigore T. Popa University of Medicine and Pharmacy Iasi, 700115 Iasi, Romania; 6 Department of Pharmacy, Faculty of Medicine, University of Prishtina, 10000 Prishtina, Kosovo; dafina.fondaj1@gmail.com; 7 Department of Pathophysiology, Forensic Veterinary Medicine and Administration, Faculty of Veterinary Medicine, University of Warmia and Mazury in Olsztyn, 10-719 Olsztyn, Poland; piotr.podlasz@uwm.edu.pl; 8 Department of Chemistry of Natural Products, Medical University of Lublin, 20-093 Lublin, Poland

**Keywords:** officinalin, stenocarpin isobutyrate, central nervous system, *Danio rerio*, hydroxycoumarins, *Peucedanum ruthenicum*

## Abstract

Anxiety is one of the most common central nervous system disorders, affecting at least one-quarter of the worldwide population. The medications routinely used for the treatment of anxiety (mainly benzodiazepines) are a cause of addiction and are characterized by many undesirable side effects. Thus, there is an important and urgent need for screening and finding novel drug candidates that can be used in the prevention or treatment of anxiety. Simple coumarins usually do not show side effects, or these effects are much lower than in the case of synthetic drugs acting on the central nervous system (CNS). This study aimed to evaluate the anxiolytic activity of three simple coumarins from *Peucedanum luxurians* Tamamsch, namely officinalin, stenocarpin isobutyrate, and officinalin isobutyrate, in a 5 dpf larval zebrafish model. Moreover, the influence of the tested coumarins on the expression of genes involved in the neural activity (*c-fos*, *bdnf*) or dopaminergic (*th1*), serotoninergic (*htr1Aa*, *htr1b*, *htr2b*), GABA-ergic (*gabarapa*, *gabarapb*), enkephalinergic (*penka*, *penkb*), and galaninergic (*galn*) neurotransmission was assessed by quantitative PCR. All tested coumarins showed significant anxiolytic activity, with officinalin as the most potent compound. The presence of a free hydroxyl group at position C-7 and the lack of methoxy moiety at position C-8 might be key structural features responsible for the observed effects. In addition, officinalin and its isobutyrate upregulated the expression of genes involved in neurotransmission and decreased the expression of genes connected with neural activity. Therefore, the coumarins from *P. luxurians* might be considered as promising drug candidates for the therapy of anxiety and related disorders.

## 1. Introduction

Anxiety disorders are characterized by excessive and unrealistic worry-like symptoms that are associated with everyday tasks or events or are specific to certain objects or rituals. Anxiety disorders are among the most frequent mental disturbances [1], affecting one-quarter of the world population and generating a broad spectrum of health problems, i.e., medical, sociological, and economic [1]. Moreover, high levels of anxiety and fear lead to avoidance of social situations and are sources of implicit and explicit costs (calculated in billions of euros per year only in Europe) including low work productivity, as well as costs associated with pharmacotherapy and psychotherapy for the affected patients [2,3].

The pathomechanism of anxiety-related disorders is complex and still not fully understood. There are several theories and concepts to explain the development of anxiety and stress states in humans. γ-aminobutyric acid (GABA) is the major inhibitory neurotransmitter in the central nervous system (CNS) involved with sedation, hypnotic, and anxiety modulation. According to monoamine theory, a disruption of the monoaminergic system in the synaptic cleft, especially involving catecholamines (dopamine and noradrenaline) and indoleamines (serotonin, 5-HT), is considered the main mechanism of anxiety disorders [4]. The majority of evidence demonstrates that under-activation of serotonergic function and complex dysregulation of noradrenergic function, toward the overactivation of this system, are implicated in emotional dysregulations [5]. Serotonin receptors 5-HT_1A,_ 5-HT_1B_, 5-HT_2A_, and 5-HT_2C_ are involved in the regulation of anxiety-related behaviors [6,7] and it has been shown that the activation of 5-HT_1A_ and 5-HT_2A_ may exert anxiolytic effects, whereas their inactivation increases anxiety-like symptoms [8]. Moreover, dopamine plays a crucial role in several brain functions and its downregulation in the CNS is involved in psychiatric and neurological disorders, including anxiety [9,10].

A group of drugs that are used clinically in the management of anxiety are benzodiazepines (BDZs). BDZs remain the first-line and the most effective choice in the treatment of anxiety disorders [11]. BDZs increase GABA affinity to the GABA_A_ receptor, elevating its binding to the receptor and therefore leading to the inhibition of neurotransmission in the neurons of the limbic system (especially the amygdala) [12]. Despite their high efficacy and the fact that they are well tolerated in short-term use, BDZs have several adverse effects, e.g., dizziness, concentration disturbances, and memory impairments [13,14]. Furthermore, long-term use of BDZs induces the rapid development of tolerance, both psychological and physical dependence, and severe withdrawal reactions [15]. Therefore, searching for new alternative substances to treat anxiety and other mental disorders is an urgent and crucial task.

Coumarins, one of the groups of natural phytochemicals, have attracted great interest as potential candidates to treat psychiatric disorders due to their versatile and readily accessible scaffold with broad-ranging biological activities [16]. Coumarins belong to a huge (more than 1300 known structures) and widely distributed class of plant derivatives, which are divided into several groups: simple coumarins, isocoumarins, biscoumarins, furanocoumarins, and pyranocoumarins, subdivided into angular and linear categories [16,17]. Their low molecular weight, relatively simple structure, high solubility in most organic solvents, multidirectional biological activity, and differences in polarity make them interesting compounds in drug discovery and development processes [16,18,19]. Their modulation of the GABA-ergic system, influence on dopamine and serotonin neurotransmission, and inhibition of acetylcholinesterase (AChE), butyrylcholinesterase (BuChE), and monoamine oxidases A and B [20] have already been reported. Due to these multidirectional activities of coumarins toward the CNS, they can exert a therapeutic effect in many psychiatric and neurodegenerative diseases, such as anxiety disorder, depression, epilepsy, and Alzheimer’s disease [20].

As nature is abundant in thousands of substances that differ in chemical structure and pharmacological activity, the establishment of a high-throughput screening activity platform, as well as an efficient method of isolation of natural compounds with high purity, seems to be necessary. Anxiety-related behavioral tests are mostly performed using rodent models so far; however, as they are time- and cost-consuming, there is a need for searching for new experimental models that will allow us to both reduce cost and investigate the complex processes occurring in the whole organism. One of the most important alternatives to rodent studies is the zebrafish (*Danio rerio*) model. Zebrafish share numerous similarities with other vertebrates in the fields of genetics and brain patterning, structure, and function in neurochemical and behavioral systems. The general organization and function of their stress-regulating system provides a high level of analogy across species [21,22]. Zebrafish larvae are highly sensitive to psychoactive drugs, e.g., diazepam, and the pharmacological effects of these substances are relatively easy to observe and measure [23,24]. One of the methods to evaluate anxiety-related behaviors is based on measuring thigmotaxis, an internal preference of animals for peripheral areas (outer zone or border zone), and avoiding the center of the open area (central zone) [25].

**Figure 1:**
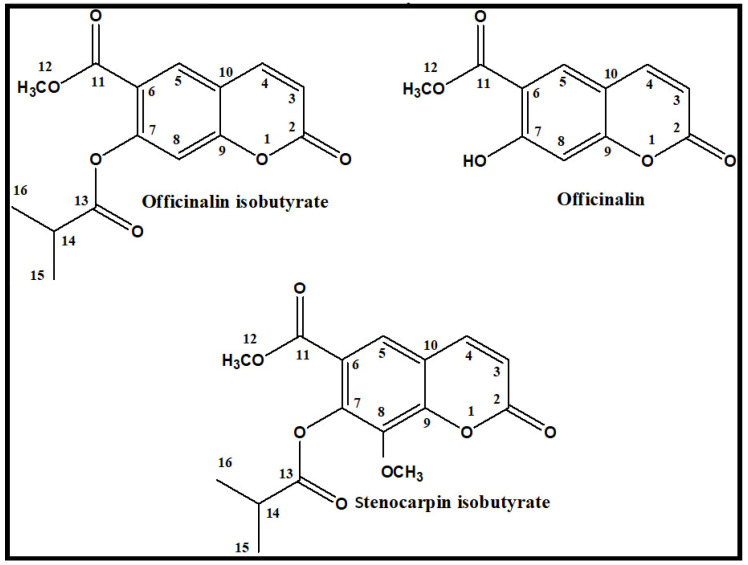
Chemical structures of isolated coumarins.

Previous studies have shown that coumarins isolated from fruits of *Seseli devenyense* Simonk. elicited pronounced anxiolytic effects in the zebrafish model of anxiety [26]. Therefore, there was a rationale for searching for other coumarin derivatives from different species from the *Apiaceae* family that exhibit activity toward the CNS. In the present study, three simple coumarins (Figure 1) (officinalin, officinalin isobutyrate, and stenocarpin isobutyrate) were selected to evaluate their influence on anxiety levels. Compounds were isolated by the countercurrent chromatography (CCC) technique from mature fruits of *Peucedanum luxurians* Tamamsch [27]. Additionally, for the first time, the influence of simple coumarins on the expression of selected genes involved in the onset and extinction of anxiety was estimated.

## 2. Results

### 2.1. The Effects of Isolated Coumarins on Thigmotaxis Behaviors of the Zebrafish Larvae during Light–Dark Changes

#### 2.1.1. The Effects of Officinalin on Locomotor Activity and Thigmotaxis Behaviors of the Zebrafish Larvae during Light–Dark Changes

**Figure 2:**
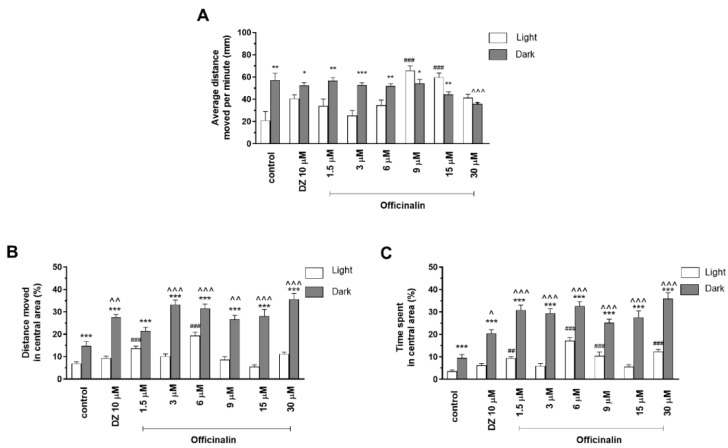
The effects of officinalin (1.5, 3, 6, 9, 15, and 30 µM) and diazepam (DZ, 10 µM) on locomotor activity during all three light–dark challenge phases. (**A**) Average distance moved by zebrafish larvae within each 1 min time bin under either light (open bars) or dark (filled bars). (**B**) The percentage of distance moved by zebrafish larvae in the central area during the light phase (white bars) or dark phase (filled bars). (**C**) The percentage of time spent by zebrafish larvae in the central area during the light phase (white bars) or dark phase (filled bars). Data are presented as mean ± SEM; n = 32. * *p* < 0.05, ** *p* < 0.01, *** *p* < 0.001 in comparison to light conditions within the same concentration group; ^ *p* < 0.05, ^^ *p* < 0.01, ^^^ *p* < 0.001 in comparison to control group under dark condition; ## *p* < 0.01, ### *p* < 0.001 in comparison to control group under light condition (post hoc Bonferroni’s test).

Two-way ANOVA showed statistically significant changes in zebrafish larvae behavior after incubation in officinalin solutions in light–dark condition response (F (1, 334) = 312.3, *p* < 0.0001), treatment effect (F (7, 334) =11.93, *p* < 0.0001), and interaction (F (7, 334) =29.48, *p* < 0.0001). The post hoc Bonferroni’s test showed an increase in locomotor activity during the dark phase in the control group (DMSO, *p* < 0.01) and with diazepam (*p* < 0.05) and officinalin at concentrations of 1.5 µM (*p* < 0.01), 3 µM (*p* < 0.001), and 6 µM (*p* < 0.01), in comparison with the light phase (Figure 2A). A decrease in locomotor activity during the dark phase was observed after incubation in officinalin at concentrations of 9 µM (*p* < 0.05) and 15 µM (*p* < 0.01), when compared to the light phase. A decrease in locomotor activity was observed only at a concentration of 30 µM (*p* < 0.001) in the dark phase in comparison with the DMSO-treated group in the dark phase (Figure 2A). During the light phase of the experiment, a significant increase in locomotor activity was noticed after incubation in officinalin at concentrations of 9 and 15 µM (*p* < 0.001), when compared to the DMSO-treated group in the light phase (Figure 2A).

Officinalin treatment in the light/dark transition influenced thigmotactic behaviors of zebrafish larvae when the distance traveled was considered (two-way ANOVA: treatment (F (7, 334) = 11.93, *p* < 0.0001), as well as interaction (F (7, 334) = 29.48, *p* < 0.0001) and light–dark condition response (F (1, 334) = 312.3, *p* < 0.0001). The post hoc Bonferroni’s test showed an increase in the percentage of distance traveled by the larvae in the inner area after DMSO (*p* < 0.001), diazepam (*p* < 0.001), and officinalin treatment in all tested concentrations (*p* < 0.001) in the dark phase of the experiment in comparison to the light phase (Figure 2B). During the dark challenge phase, a significant increase in the percentage of the distance traveled was observed when the 5 dpf larvae were treated with diazepam (*p* < 0.01) and officinalin at concentrations of 3, 6, 15, and 30 µM (*p* < 0.001) as well as 9 µM (*p* < 0.01) as compared with the DMSO-treated group in the dark phase (Figure 2B). During the light phase of the experiment, a significant increase in the percentage of the distance traveled in the central area was noticed when 5 dpf larvae were treated with officinalin at concentrations of 1.5 and 6 µM (*p* < 0.001), when compared to the DMSO-treated group in the light phase (Figure 2B).

Officinalin at the tested concentrations also demonstrated a significant influence on thigmotactic behaviors of 5 dpf larvae, when the duration of the activities in the central area was considered (two-way ANOVA: treatment (F (7, 334) = 6.847, *p* < 0.0001), as well as interaction (F (7, 334) = 20.71, *p* < 0.0001) and light–dark condition response (F (1, 334) = 174.2, *p* < 0.0001). The post hoc test (Bonferroni’s test) demonstrated an increase in the percentage of time spent in the central area during the dark phase of the experiment after DMSO (*p* < 0.001), diazepam (*p* < 0.001), and officinalin treatment at all tested concentrations (1.5; 3; 6; 9; 15; and 30 µM; *p* < 0.001) compared with the light phase (Figure 2C). Moreover, during the dark phase of the experiment, a significant increase in duration in the central area was noticed when the larvae were treated with diazepam (*p* < 0.05), as well as all concentrations of officinalin (*p* < 0.001), as compared with the DMSO-treated group in the dark phase (Figure 2C). During the light phase of the experiment, a significant increase in the percentage of time spent in the central area was noticed when 5 dpf larvae were treated with officinalin at concentrations 1.5 µM (*p* < 0.01) as well as 6, 9, and 30 µM (*p* < 0.001), when compared to the DMSO-treated group in the light phase (Figure 2C).

#### 2.1.2. The Effects of Stenocarpin Isobutyrate on Locomotor Activity and Thigmotaxis Behaviors of the Zebrafish Larvae during Light–Dark Changes

**Figure 3:**
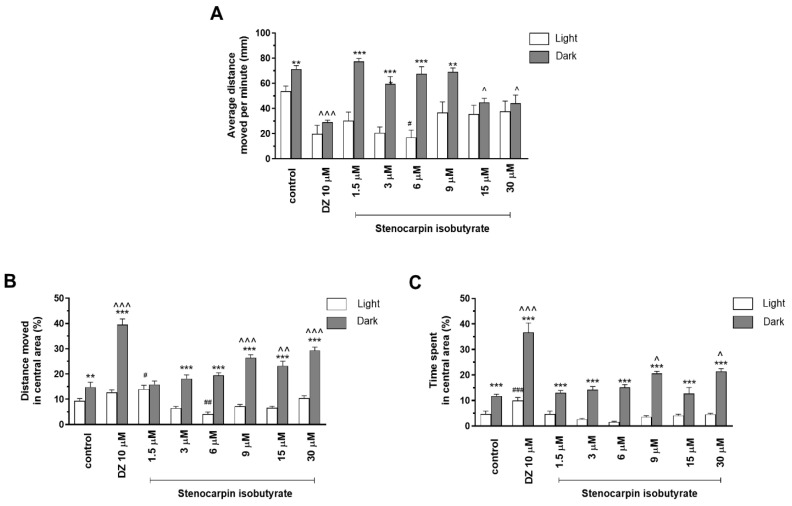
The effects of stenocarpin isobutyrate (1.5, 3, 6, 9, 15, and 30 µM) and diazepam (DZ, 10 µM) on locomotor activity during all three light–dark challenge phases. (**A**) Average distance moved by zebrafish larvae within each 1 min time bin under either light (open bars) or dark (filled bars). (**B**) The percentage of distance moved by zebrafish larvae in the central area during the light phase (white bars) or dark phase (filled bars). (**C**) The percentage of time spent by zebrafish larvae in the central area during the light phase (white bars) or dark phase (filled bars). Data are presented as mean ± SEM; n = 32. ** *p* < 0.01, *** *p* < 0.001 in comparison to light conditions within the same concentration group; ^ *p* < 0.05, ^^ *p* < 0.01, ^^^ *p* < 0.001 in comparison to control group under dark condition; # *p* < 0.05, ## *p* < 0.01, ### *p* < 0.001 in comparison to control group under light condition (post hoc Bonferroni’s test).

Two-way ANOVA showed statistically significant changes in zebrafish larvae behavior after incubation in stenocarpin isobutyrate solutions in light–dark condition response (F (1, 334) = 15.345, *p* = 0.0001), treatment effect (F (7, 334) = 51.90, *p* = 0.0001), and interaction (F (7, 334) = 6.615, *p* < 0.0001). The post hoc Bonferroni’s test showed an increase in locomotor activity during the dark phase in the control group (DMSO, *p* < 0.01) and with stenocarpin isobutyrate at concentrations of 1.5, 3, and 6 µM (*p* < 0.001) as well as 9 µM (*p* < 0.01), in comparison with the light phase (Figure 3A). A decrease in locomotor activity during the dark phase was observed after incubation in diazepam (*p* < 0.001) and stenocarpin isobutyrate at concentrations of 15 and 30 µM (*p* < 0.05) in comparison with the DMSO-treated group in the dark phase (Figure 3A). During the light phase of the experiment, a significant decrease in the locomotor activity was noticed after incubation in stenocarpin isobutyrate at concentrations of 6 µM (*p* < 0.05), when compared to the DMSO-treated group in the light phase (Figure 3A).

Stenocarpin isobutyrate treatment in the light/dark challenge also had an impact on thigmotactic behaviors of 5 dpf larvae when the distance traveled was considered (two-way ANOVA: treatment (F (7, 334) = 11.93, *p* < 0.0001), as well as interaction (F (7, 334) = 29.48, *p* < 0.0001) and light–dark condition response (F (1, 334) = 312.3, *p* < 0.0001). The post hoc Bonferroni’s test showed an increase in the percentage of distance traveled by zebrafish larvae in the inner area after DMSO (*p* < 0.01), diazepam (*p* < 0.001), and stenocarpin isobutyrate treatments at concentrations of 3, 6, 9, 15, and 30 µM (*p* < 0.001) in the dark phase of the experiment in comparison to the light phase (Figure 3B). During the dark challenge phase, a significant increase in the percentage of distance traveled was observed when the 5 dpf larvae were treated with diazepam (*p* < 0.001) and stenocarpin isobutyrate at concentrations of 9 and 30 µM (*p* < 0.001) as well as 15 µM (*p* < 0.01) when compared with the DMSO-treated group in dark phase (Figure 3B). During the light phase of the experiment, a significant increase in the percentage of the distance traveled in the central area was noticed when 5 dpf larvae were treated with officinalin at a concentration of 1.5 µM (*p* < 0.05), and a decrease was observed after incubation at the concentration of 6 µM (*p* < 0.01) when compared to the DMSO-treated group in the light phase (Figure 3B).

Stenocarpin isobutyrate at the tested concentrations also demonstrated a significant influence on thigmotactic behaviors of 5 dpf larvae, when the duration of the activities in the central area was considered (two-way ANOVA: treatment (F (7, 334) = 6.847, *p* < 0.0001), as well as interaction (F (7, 334) = 20.71, *p* < 0.0001)) and light–dark condition response (F (1, 334) = 174.2, *p* < 0.0001). The post hoc Bonferroni’s test showed an increase in the percentage of time spent in the central area during the dark phase of the experiment after DMSO (*p* < 0.001), diazepam (*p* < 0.001), and stenocarpin isobutyrate treatment at all tested concentrations (1.5; 3; 6; 9; 1.5; and 30 µM) compared with the light phase (Figure 3C). Moreover, during the dark phase of the experiment, a significant increase in duration in the central area was noticed when the larvae were treated with diazepam (*p* < 0.001) and stenocarpin isobutyrate at concentrations of 9 and 30 µM (*p* < 0.05) compared with the DMSO-treated group in the dark phase (Figure 3C). During the light phase of the experiment, a significant increase in the percentage of time spent in the central area was noticed when 5 dpf larvae were treated with diazepam (*p* < 0.001) when compared to the DMSO-treated group in the light phase (Figure 3C).

#### 2.1.3. The Effects of Officinalin Isobutyrate on Locomotor Activity and Thigmotaxis Behaviors of the Zebrafish Larvae during Light–Dark Changes

**Figure 4:**
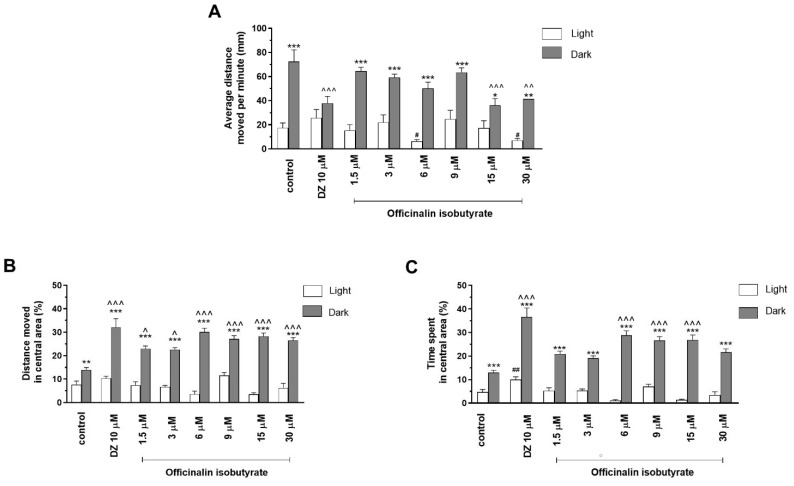
The effects of officinalin isobutyrate (1.5, 3, 6, 9, 15, and 30 µM) and diazepam (DZ, 10 µM) on locomotor activity during all three light–dark challenge phases. (**A**) Average distance moved by zebrafish larvae within each 1 min time bin under either light (open bars) or dark (filled bars). (**B**) The percentage of distance moved by zebrafish larvae in the central area during the light phase (white bars) or dark phase (filled bars). (**C**) The percentage of time spent by zebrafish larvae in the central area during the light phase (white bars) or dark phase (filled bars). Data are presented as mean ± SEM; n = 32. * *p* < 0.05, ** *p* < 0.01, *** *p* < 0.001 in comparison to light conditions within the same concentration group; ^ *p* < 0.05, ^^ *p* < 0.01, ^^^ *p* < 0.001 in comparison to control group under dark condition; # *p* < 0.05; ## *p* < 0.01 in comparison to control group under light condition (post hoc Bonferroni’s test).

Two-way ANOVA showed statistically significant changes in zebrafish larvae behavior after incubation in officinalin isobutyrate solutions in light–dark condition response (F (1, 344) = 136.5, *p* < 0.0001), treatment effect (F (7, 2344) = 4.297, *p* = 0.0002), and interaction (F (7, 344) = 5.81, *p* < 0.0001). The post hoc Bonferroni’s test showed an increase in the locomotor activity during the dark phase in the control group (DMSO, *p* < 0.001) and with officinalin isobutyrate at concentrations of 1.5 µM, 3 µM, 6 µM, 9 µM (*p* < 0.001), 15 µM (*p* < 0.05), and 30 µM (*p* < 0.01), in comparison with the light phase (Figure 4A). A decrease in locomotor activity during the dark phase was observed after incubation in diazepam (*p* < 0.001) and after administration of officinalin isobutyrate at the concentrations of 15 µM (*p* < 0.001) and 30 µM (*p* < 0.01), in comparison with the DMSO-treated group in the dark phase (Figure 4A). During the light phase of the experiment, a significant decrease in locomotor activity was noticed after incubation with officinalin isobutyrate at concentrations of 6 µM and 30 µM (*p* < 0.05), as compared to the DMSO-treated group in the light phase (Figure 4A).

Treatment with officinalin isobutyrate during the light/dark challenge had an impact on the thigmotactic behavior of *Danio rerio* larvae, as assessed by the distance traveled in the central arena (two-way ANOVA: interaction (F (7, 344) = 10.44, *p* < 0.0001), light–dark condition response, (F (1, 344) = 409.6, *p* < 0.0001), and treatment effect (F (7, 344) = 5.170, *p* < 0.0001). The post hoc Bonferroni’s test revealed an increase in the percentage of the distance traveled by the 5 dpf larvae in the central arena (inner zone) after incubation in DMSO (control, *p* < 0.01), diazepam (*p* < 0.001), and officinalin isobutyrate at all used concentrations (*p* < 0.001) in the dark phase as compared with the light phase (Figure 4B). In addition, during the dark phase of the experiment, significant increases in the distance traveled in the central area were observed when 5 dpf larvae were treated with diazepam (*p* < 0.001) and officinalin isobutyrate at concentrations of 1.5 and 3 µM (*p* < 0.05) and 6, 9, 15, and 30 µM (*p* < 0.001) as compared with the DMSO-treated group in the dark phase (Figure 4B). 

Officinalin isobutyrate in the light–dark challenge also influenced the thigmotactic behavior of zebrafish larvae when the time spent in the central arena (expressed in %) was considered (two-way ANOVA: treatment (F (7, 344) = 12.62, *p* < 0.0001), as well as interaction (F (7, 344) = 12.14, *p* < 0.0001) and light–dark condition response (F (1, 344) = 549.9, *p* < 0.0001). The post hoc Bonferroni’s test revealed an increase in the percentage of the time spent by the 5 dpf larvae in the central arena (inner zone) after incubation in DMSO (control, *p* < 0.001), diazepam (*p* < 0.001), and officinalin isobutyrate at all used concentrations (*p* < 0.001) in the dark phase as compared with the light phase (Figure 4C). In addition, during the dark phase of the experiment, significant increases in time spent in the central area were observed when 5 dpf larvae were treated with diazepam (*p* < 0.001) and officinalin isobutyrate at concentrations of 6, 9, and 15 µM (*p* < 0.001) as compared with the control group (DMSO-treated) (Figure 4C). During the light phase of the experiment, a significant increase in the percentage of time spent in the central arena was observed with treatment with diazepam (*p* < 0.01) when compared to the DMSO-treated group in the light phase (Figure 4C).

### 2.2. Influence of Isolated Coumarins on the Expression of Genes Associated with the Etiology and Extinction of Anxiety

The effects of diazepam (10 µM) and tested coumarins (officinalin, stenocarpin isobutyrate, and officinalin isobutyrate, 6 µM) on the level of expression of 11 genes associated with anxiety were determined by RT PCR. The doses were selected based on the results of behavioral tests. 

**Figure 5:**
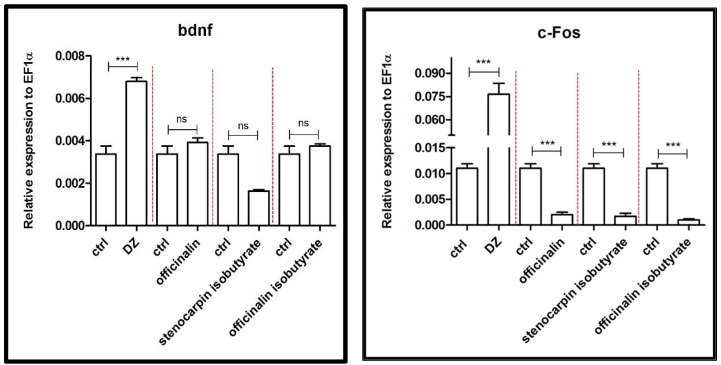
Expression profiles of *bdnf* and *c-fos* genes associated with neuronal activity after exposure to 10 µM concentration of diazepam and 6 µM concentration of tested coumarins. Data are presented as mean ± SEM; *** *p* < 0.001 in comparison to the control group.

Results presented in Figure 5 show that acute administration of coumarins had no significant influence on the expression of the *bdnf* gene, in opposite to diazepam, which significantly increased the level of expression (*p* < 0.001) of *bdnf* in the zebrafish larvae compared to the control group.

Furthermore, diazepam significantly increased the expression of *c-fos* (*p* < 0.001), whereas coumarins significantly decreased the expression of this gene (*p* < 0.001) (Figure 5).

**Figure 6:**
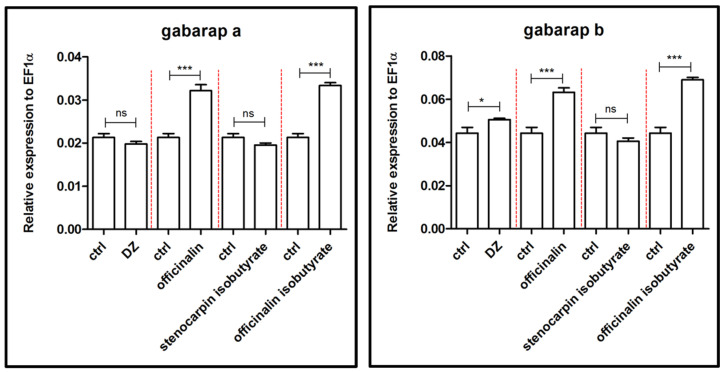
Expression profiles of *gabarapa* and *gabarapb* genes associated with GABA-ergic system after exposure to 10 µM concentration of diazepam and 6 µM concentration of tested coumarins. Data are presented as mean ± SEM; * *p* < 0.05; *** *p* < 0.001 in comparison to the control group.

An increased expression of genes responsible for GABA-receptor-associated proteins a and b (*gabarapa* and *gabarapb*) was observed after treatment of 5 dpf larvae with officinalin and its isobutyrate (*p* < 0.001) in comparison to the control group. Stenocarpin isobutyrate did not exert a significant effect (Figure 6). Diazepam altered expression only in the case of the *gabarapb* gene (*p* < 0.05).

**Figure 7:**
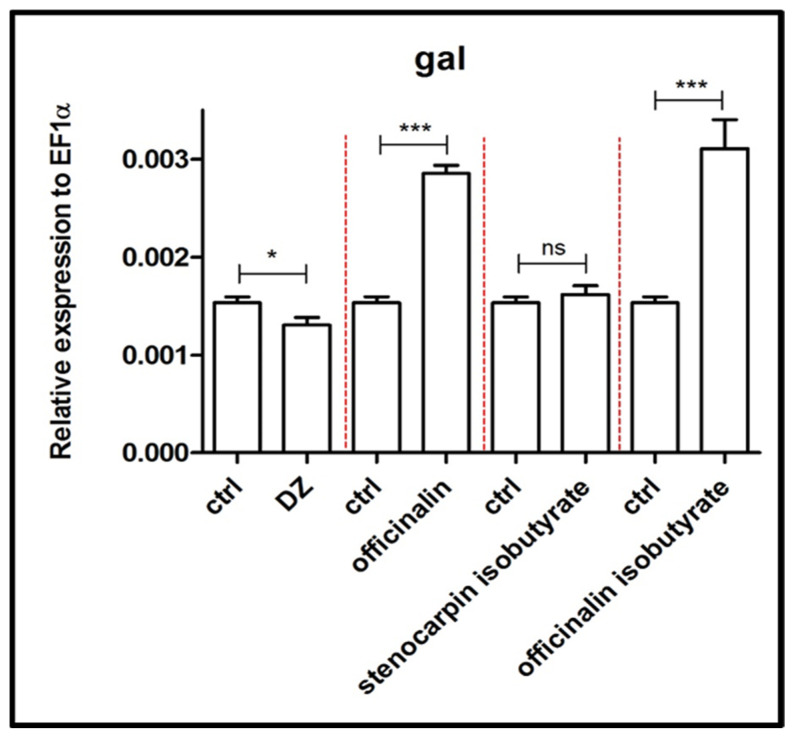
Expression profile of *gal* gene associated with galaninergic system after exposure to 10 µM concentration of diazepam and 6 µM concentration of tested coumarins. Data are presented as mean ± SEM; * *p* < 0.05; *** *p* < 0.001 in comparison to the control group.

As depicted in Figure 7, expression of the *gal* gene was strongly increased by treatment with officinalin and officinalin isobutyrate (*p* < 0.001) as compared to the control group. Diazepam reduced the level of expression of the *gal* gene (*p* < 0.05), whereas stenocarpin isobutyrate had no statistically significant influence on this process.

**Figure 8:**
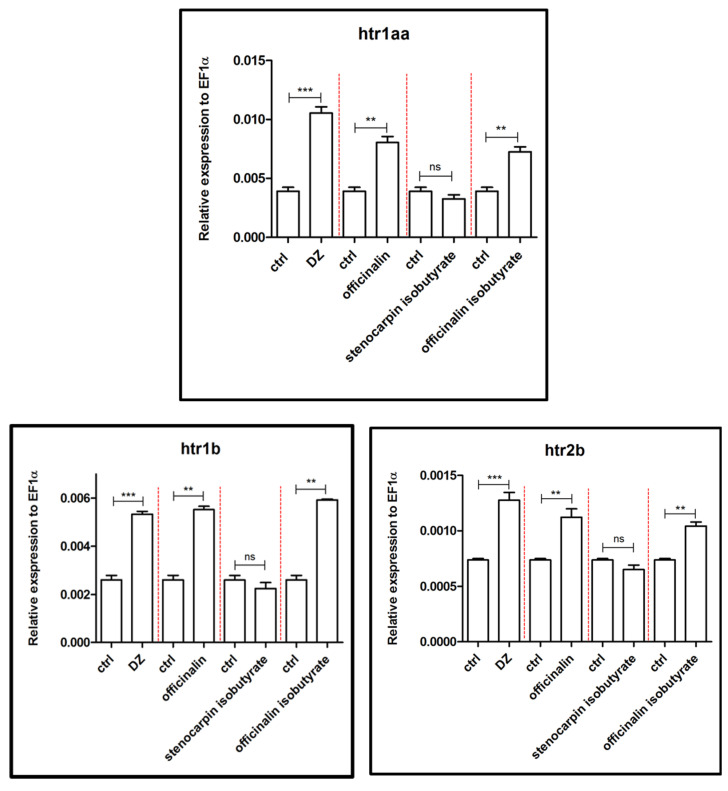
Expression profiles of *htr1Aa*, *htr1b*, and *htr2b* genes associated with the serotoninergic system after exposure to 10 µM concentration of diazepam and 6 µM concentration of tested coumarins. Data are presented as mean ± SEM; ** *p* < 0.01; *** *p* < 0.001 in comparison to the control group.

The acute administration of officinalin and officinalin isobutyrate at a concentration of 6 µM on 5 dpf zebrafish larvae significantly increased (*p* < 0.01) the expression of three genes related to the serotoninergic system (*htr1aa*, *htr1b,* and *htr2b*) in comparison to the control group (Figure 8). The strongest influence on the expression of these genes was observed after treatment of 5 dpf zebrafish larvae with 10 µM diazepam (*p* < 0.001), in comparison to the control group. Stenocarpin isobutyrate did not exert a significant effect on *htr1aa*, *htr1b,* and *htr2b* gene expression.

**Figure 9:**
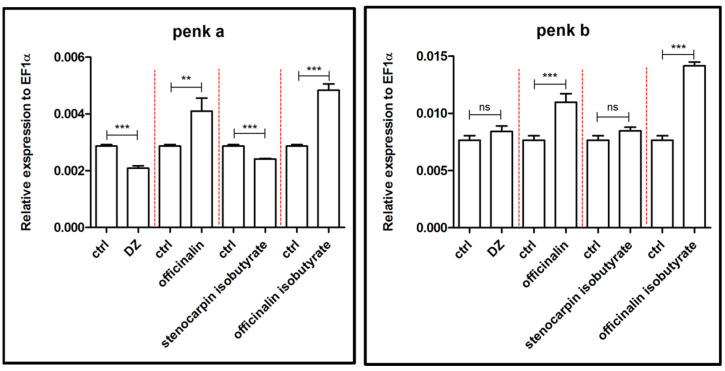
Expression profiles of *penka* and *penkb* genes associated with the enkephalinergic system after exposure to 10 µM concentration of diazepam and 6 µM concentration of tested coumarins. Data are presented as mean ± SEM; ** *p* < 0.01; *** *p* < 0.001 in comparison to the control group.

In Figure 9, the effects of officinalin, stenocarpin isobutyrate, and officinalin isobutyrate (6 µM), as well as 10 µM diazepam, on the expression of the genes *penka* and *penkb,* related to the enkephalinergic system, are shown. Officinalin isobutyrate enhanced the expression of both genes in a significant manner (*p* < 0.001), whereas officinalin elevated the level of expression of *penkb* (*p* < 0.001) more than *penka* (*p* < 0.01) when compared to the control group. Moreover, stenocarpin isobutyrate and diazepam decreased significantly the expression of the *penka* gene (*p* < 0.001), whereas both compounds did not influence the expression of the *penkb* gene when compared to the control group.

**Figure 10:**
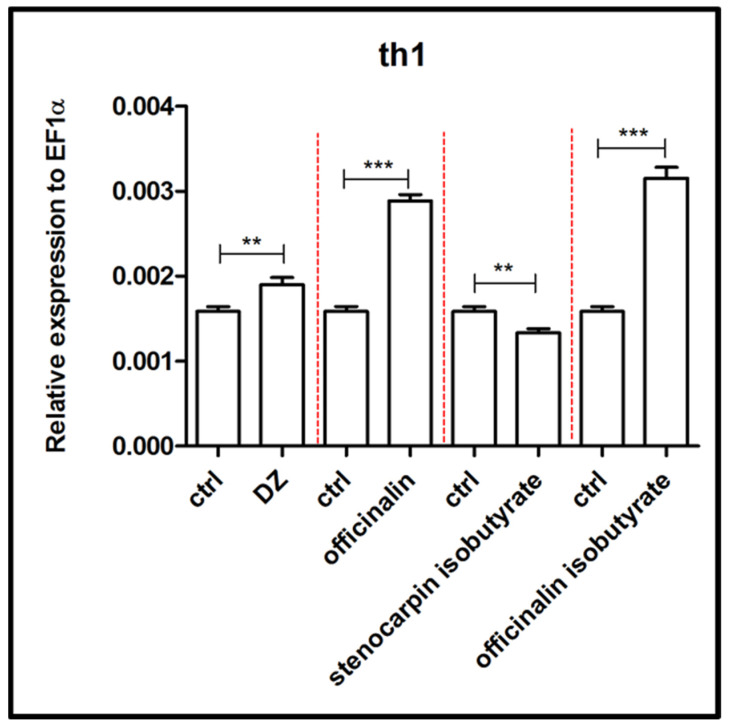
Expression profiles of *th1* genes associated with the dopaminergic system after exposure to 10 µM concentration of diazepam and 6 µM concentration of tested coumarins. Data are presented as mean ± SEM; ** *p* < 0.01; *** *p* < 0.001 in comparison to control group.

An increased expression of genes responsible for encoding tyrosinase hydroxylase (*th1)* was observed after treatment of 5 dpf larvae with officinalin and its isobutyrate (*p* < 0.001), as well as diazepam (*p* < 0.01), in comparison to the control group (Figure 10). Stenocarpin isobutyrate reduced the expression of the *th1* gene (*p* < 0.01), compared to the control group.

During the light conditions, the tested compounds had no effect on locomotor activity; therefore, the further-described results were not ascribed to an impairment in the locomotor activity of the zebrafish. Nonetheless, results are shown as Supplementary files (Figures S1–S3). Furthermore, officinalin had no influence on thigmotactic behavior during the 40 min light phase of the experiment (concerning both distance moved and time spent in the central area) (Figure S1b,c). The other tested compounds (stenocarpin isobutyrate and officinalin isobutyrate) did not show any effect on the distance traveled in the central area during the 40 min light phase of the experiment (Figures S2b and S3b). Stenocarpin isobutyrate at concentrations of 1.5 and 3 µM (Figure S2c) and officinalin isobutyrate at concentrations of 1.5–15 µM (Figure S3c) did not exert an effect on time spent in the central area during the 40 min light phase of the experiment.

## 3. Discussion

The presented study showed the effects of the three simple coumarins on anxiety-related behavior in 5 dpf zebrafish larvae. Overall, officinalin, officinalin isobutyrate, and stenocarpin isobutyrate increased the percentage of time (spent) and distance (moved) in the inner (central) area during the dark phase in comparison with the light phase. It is worth mentioning that during the continuous light condition (Figures S1–S3), no tested compound demonstrated any influence on the locomotor activity of the larvae.

The difference in the anxiolytic activity between the three coumarin derivatives could be related to several chemical features, such as the presence of a free hydroxyl group at position C-7 and the lack of methoxy moiety at position C-8 in the structure of officinalin, the most potent compound. Esterification at C-7 with isobutyric acid (2-methyl propanoic acid) slightly reduced the activity, whereas an additional methoxy group at C-8 reduced significantly the anxiolytic activity of stenocarpin isobutyrate. 

Data drawn from the literature indicate the anti-anxiety activity of simple coumarins [28,29], especially those with a 7-hydroxycoumarin scaffold, such as umbelliferone [28], ostruthin [29], daphnoretin [30], or devenyol [26]. Results concerning the anxiolytic activity of all these coumarins might indicate that the free hydroxyl group at position C7 is probably crucial for their anxiolytic effects [26]. 

Moreover, our results show the compound-specific effects of the tested coumarins on the changes in anxiety-related gene expression. The GABA-ergic system is crucial for anxiety development, and the same applies for anxiolytic activity [31]. Officinalin and its isobutyrate altered the expression of *gabarapa* and *gabarapb* genes, responsible for coding GABA_A_-receptor-associated proteins a and b. The *Danio rerio gabrapa* and *gabarapb* are homologous to human GAPARAP [32]. There is compelling evidence that co-expression of GABARAP with subunits of GABA_A_ receptors results in increased clustering of receptors in many cells [33]. These observations suggest that GABARAP may be involved at an early stage in the clustering of GABA_A_ receptors, even though it appears not to be present as part of the anchoring mechanism for the receptors in the cell membrane [34]. 

Galanin is widely expressed throughout the CNS, as well as peripherally [35]. In the brain, galanin may act as an inhibitory neuromodulator and hypophysiotropic messenger in the anterior pituitary [36]. Officinalin and its isobutyrate increased the relative expression of *galn*, the gene responsible for coding galanin, whereas stenocarpin isobutyrate was inactive. Moreover, these findings correspond with research concerning the changes in the galanin system in a rat model of PTSD, indicating that a reduction in galanin expression may contribute to anxiety-like behavior, whereas its increased expression may play a role in a compensatory response to counteract the increased anxiety mediated by other neurotransmitters [37]. Furthermore, the same two coumarins might modulate the serotoninergic system by enhancing the relative expression level of three genes coding serotoninergic receptors 5HT_1A_, 5HT_2A,_ and 5HT_2B_, namely *htr1a*, *htr1b,* and *htr2b*. Studies in animal models have suggested that anxiety-like behavior can increase when the function of HTR1A (the orthologous human and rodent gene) is eliminated or overexpressed [38].

Enkephalins are endogenous opioid peptides that are derived from pro-prenkephalins precursor proteins [39]. In zebrafish, two genes are responsible for coding proenkepalins: *penka* (for proenkephalin a) and *penkb* (for proenkephalin b); *penka and penkb* zebrafish genes are orthologous to rodent Penk and human PENK genes [40]. PENK neurons are widespread throughout the CNS as well as the peripheral nervous system (PNS). Numerous PENK neurons occur in limbic system structures; for example, the hippocampus, septum, and bed nucleus of the stria terminalis (BNST) [41]. Considering the location of its neurons in the brain structure, the opioid system plays a crucial role in the neural modulation of anxiety [42]. Modulation of anxiety-like behavior by the opioid system has been confirmed in the zebrafish model [40]. In our study, officinalin and officinalin isobutyrate showed a significant effect on increasing the relative expression of the *penka* gene and *penkb* gene. In contrast, stenocarpin isobutyrate decreased the relative expression level of the *penka* gene, without influence on *penkb* expression.

In addition, the influence on the relative expression level of the *th1* gene, responsible for coding the TH enzyme and involved in the synthesis of catecholamines, was also evaluated. Officinalin and its isobutyrate were the most active coumarins, whereas stenocarpin isobutyrate decreased the level of the relative expression of *th1*. Tu and co-workers confirmed that the disruption in serotonin, dopamine, and GABA neurotransmission pathways caused by tributyltin, which results in enhanced anxiety responses in zebrafish males, was caused by significant inhibition of the expressions of genes (among them *th1*) responsible for the synthesis and action of neurotransmitters [43]. Thus, our study also confirmed the involvement of an increased expression level of the *th1* gene in anxiolytic behaviors. 

Finally, the influence on *c-fos* and *bdnf,* genes involved in the synthesis of the protein related to the regulation of neural activity, was assessed. All tested simple coumarins downregulated the relative expression of zebrafish *c-fos* and proto-gene coding c-fos protein, without influence on *bdnf* expression. *c-fos* mRNA is found in low levels in the normal physiological state in the adult mammalian CNS [44]. However, *c-fos* is an early induction gene, and its expression is rapidly and transiently induced in neurons because of a variety of exogenous and endogenous factors that cause changes that disrupt the body’s homeostasis. Therefore, the expression level of *c-fos* has been considered an excellent marker of neuronal activity [36,45]. Brain-derived neurotrophic factor (BDNF) is broadly expressed in the adult mammalian brain, as well as its receptor tyrosine receptor kinase B (TrkB) [46]. In humans and rodents, decreasing hippocampal BDNF concentrations may be involved in the onset of depression and anxiety, with implications regarding the hippocampus, which is considered to be involved in learning, mood, and anxiety, as a port of the limbic system [47]. The results obtained in our research showed the differences in these genes’ expression under the influence of diazepam and coumarins, although the behavioral effect was not significant. Further studies should be performed to explain these discrepancies.

## 4. Materials and Methods

### 4.1. Drugs

The following compounds were used during experiments: diazepam (Sigma-Aldrich, St. Louis, MO, USA); officinalin isobutyrate (methyl 7-(2-methylpropanoyloxy)-2-oxochromene-6-carboxylate); officinalin (methyl 7-hydroxy-2-oxo-2*H*-1-benzopyran-6-carboxylate); and stenocarpin isobutyrate (methyl 8-methoxy-7-(2-methyl-1-oxopropoxy)-2-oxo-2*H*-1-benzopyran-6-carboxylate). All coumarins were isolated from mature fruits of *Peucedanum luxurians* Tamamsh. (syn. *Peucedanum ruthenicum* M.Bieb.; Apiaceae) according to the previously described method [27]. Fruits were air-dried and pulverized, and extraction was performed by the accelerated solvent extraction method (ASE) using the Dionex ASE 100 instrument (Dionex, Sunnyvale, CA, USA). Dichloromethane was used as an extraction solvent. Three tested coumarins were isolated from the extract in a high-performance counter-current chromatograph (HPCCC). The identity of investigated coumarins was confirmed by HPLC-DAD-ESI-Q-TOF-MS and 1H NMR analyses.

### 4.2. Zebrafish Model for Evaluation of Anxiety-Like Behaviors

#### 4.2.1. Animal Husbandry

Zebrafish (*Danio rerio*) stocks of the AB strains were maintained at 28.5 C, on a 14/10 h light/dark cycle under the standard aquaculture conditions. Fertilized eggs were collected via natural spawning. Embryos were bred in E3 embryo medium (pH 7.1–7.3; 17.4 µM NaCl, 0.21 µM KCl, 0.12 µM MgSO_4_, and 0.18 µM Ca(NO_3_)_2_) in an incubator (IN 110 Memmert GmbH, Buechenbach, Germany) with a 14/10 h light/dark cycle. In these experiments, 5 days post fertilization (dpf), zebrafish larvae were used for the assays. After the experiments, larvae were immediately euthanized by immersion in a solution of tricaine (15 μM). All experiments were conducted in accordance with the National Institute of Health Guidelines for the Care and Use of Laboratory Animals and with the European Community Council Directive for the Care and Use of Laboratory Animals of 22 September 2010 (2010/63/EU).

#### 4.2.2. Determination of the Maximum Tolerated Concentration

In the first step of research, the maximum tolerated concentration (MTC) for each compound was determined. Zebrafish larvae were incubated 4 dpf for 18 h in a dark environment at 28 °C with the tested compounds in a gradient of 4 concentrations—12.5, 25, 50, and 100 μM in 48-well plates; 0.5 mL of the solution to each well. In each of the wells, 5 larvae were placed. In total, 10 larvae per concentration were tested. The MTC was selected based on the lack of occurrence of the following symptoms: hypoactivity, no touch response, decreased touch response, loss of posture, body deformation, exophthalmos, slow heartbeat, edema, and death. Based on preliminary studies, the concentrations of 1.5, 3, 6, 9, 15, 30 µM were selected (the toxicity was exhibited starting from the dose of 50 μM) for further anxiety-related experiments. As a negative control, 1% DMSO in the E3 solution was used. Diazepam—positive control—was tested at an anxiolytic dose of 10 µM; the dose was selected based on our previous studies [48].

#### 4.2.3. Anxiolytic Activity Assay

The experimental procedure was based on our previous studies [26], wherein we used procedures established by Peng et al. [49]. The anxiolytic activity assay was performed involving 5 dpf larvae in 24-well plates (one larva in each well) with 1.5 mL of freshly prepared solution. Larvae were exposed to the tested coumarins 30 min before the test.

Zebrabox (Viewpoint, Lyon, France) with ZebraLab software was used for video tracking. The experiment duration was 95 min, with the first 10 min of acclimatization followed by 40 min of continuous light for studying spontaneous locomotor activity. Then, fish were exposed to three light–dark transitions (15 min each cycle consisting of 10 min of illumination followed by 5 min of darkness), which triggered anxiety-like behavior. The outer and central (inner) zones of the wells were marked to enable thigmotaxis measurement. The measured parameters were the time spent and distance moved in the inner zone in comparison to time and distance in total area (outer and inner zone). The results were presented as the percentage of time spent and distance moved in the central area.

### 4.3. RNA Isolation and Quantitative PCR

Immediately after exposure to anxiety-provoking conditions, larvae of each group were polled (n = 30) and frozen (−80 °C). Total RNA was extracted using the Total RNA Mini isolation kit (AA Biotechnology, Gdynia, Poland) in accordance with the manufacturer’s protocols. cDNA required for qPCR analyses was synthesized from normalized RNA concentrations using a Maxima First Strand cDNA Synthesis Kit for RT-qPCR (Thermo Scientific, Waltham, MA, USA). The reverse transcription process was carried out according to the manufacturer’s protocols. qPCR was performed using SYBR Green (SYBR Select Master Mix, Applied Biosystems, Foster City, CA, USA) on a 7500 Fast Real-Time PCR System instrument (Applied Biosystems, Foster City, CA, USA) under previously described conditions [50]. Oligonucleotide primers selected to detect genes involved in anxiety response are listed in Table 1. Initial validation of reference genes revealed that for the purpose of the study, *elongation factor 1-alpha (ef1-α)* showed the most efficient and equal expression among the samples. The values of the expression of the studied genes were calculated in each group as an expression relative to *Ef1-α*. Each sample was analyzed in triplicate in three separate experiments.

### 4.4. Statistical Analysis

For the determination of the statistical differences between various tested substances and their concentrations, Prism software (GraphPad Software, San Diego, CA, USA) was used. Data were presented as mean ± standard error of the mean (SEM). For comparison, the pretreatment- and treatment-effects obtained data were analyzed using analysis of variance (one-way or two-way ANOVA). One-way ANOVA was followed by a Tukey’s test (post hoc test). In the case of two-way ANOVA, Bonferroni’s test was used as a post hoc test. The confidence limit of *p* < 0.05 was considered statistically significant.

## 5. Conclusions

Our study shows the anxiolytic activity of coumarins in behavioral tests as well as their influence on the expression of genes connected with anxiety onset and its extinction, using the zebrafish model. Here, we screened the activity of three coumarins with different chemical structures (i.e., officinalin, stenocarpin isobutyrate, and officinalin isobutyrate) isolated from the fruits of *Peucedanum luxurians* Tamamsh. We observed that all compounds exhibited anti-anxiety effects, although officinalin and its isobutyrate demonstrated the most prominent activity. The behavioral effects stayed in line with the gene expression; *gaba a, gabab, gal, htr1aa, htr1b, htr2b* and *penka* were modulated only after the exposure of zebrafish to officinalin and its isobutyrate. The presence of a free hydroxyl group at position C-7 and the lack of methoxy moiety at position C-8 could be important for the anxiolytic effect.

The current study demonstrates the usefulness of zebrafish as a model organism for screening natural products with potential anxiolytic activity, evaluating gene expression, and identifying chemical features required for anxiolytic effects.

## Figures and Tables

**Table 1:** Primers used in the study.

Gene	Forward 5′-3′	Reverse 5′-3′	Accession No/Source
*c-fos*	GTGCAGCACGGCTTCACCGA	TTGAGCTGCGCCGTTGGAGG	**NM_205569.1**
*th1*	GACGGAAGATGATCGGAGACA	CCGCCATGTTCCGATTTCT	**NM_131149.1/**
*gal*	GACCAACTGATACTCAGGATGCA	ATCCCGAGTGTTTCTGTCAGAA	**NM_001346239.1**
*gabrapa*	AGGATCCACTTGAGGGCTGA	CTTCTTCGTGATGCTCCTGGT	**NM_001013260.1/**
*gabarapb*	TGTAAACAACGTCATTCCCCCT	ATCTTCTTCGTGATGTTCCTGGT	**NM_001386387.1**
*penka*	CTTTGAGCGCCTGTCTCGTG	TTCTAATGTGCAGGCCAGTGTG	**NM_200083.2**
*penkb*	TGGTGGCAGGAGTCTAAACG	TTGGACATCGCGGACATCAT	**NM_182883.1**
*htr1aa*	CAGAGCAGAGCAGCACAAG	TGGTCTGAGAGTTCTGGTCTAATC	**NM_001123321.1**
*htr1b*	GTGTCGGTGCTCGTGATG	CAGCCAGATGTCGCAGATG	**NM_001128709.1**
*htr2b*	GCTGCTCATTCTTCTGGTCAT	GTTAGTGGCGTTCTGGAGTT	**NM_001044743.1**
*bdnf*	GGCGAAGAGCGGACGAATATC	AAGGAGACCATTCAGCAGGACAG	**NM_131595.2**
*Ef1α*	CTGGAGGCCAGCTCAAACAT	ATCAAGAAGAGTAGTACCGCTAGCATTAC	**NM_131263.1**

## Data Availability

Not applicable.

## Supplementary Materials

The following supporting information can be downloaded at: https://www.mdpi.com/article/10.3390/ijms24108693/s1.
