# Supplementary material for: Simple Coumarins from Peucedanum luxurians Fruits: Evaluation of Anxiolytic Activity and Influence on Gene Expression Related to Anxiety in Zebrafish Model
Source: Int J Mol Sci. 2023 May 12;24(10):8693. doi: 10.3390/ijms24108693 (PMC10218424; doi:10.3390/ijms24108693)
Supplement: Supplementary file 1 [file ijms-24-08693-s001.zip › ijms-2327645-supplementary.pdf]

International Journal of *Molecular Sciences*

MDPI

Article

Simple Coumarins from *Peucedanum luxurians* Fruits: Evaluation of Anxiolytic Activity and Influence on Genes Expression Related to Anxiety on Zebrafish Model

Jarosław Widelski [1,*], Natalia Kasica [2], Monika Maciąg [3], Simon Vlad Luca [4,5], Barbara Budzyńska [3], Dafina Fondai [6], Piotr Podlasz [7] and Krystyna Skalicka-Woźniak [8,*]

1 Department of Pharmacognosy with Medicinal Plant Unit, Medical University of Lublin, 20-093 Lublin, Poland; jwidelski@pharmacognosy.org (J.W.)
2 Department of Animal Anatomy, Faculty of Veterinary Medicine, University of Warmia and Mazury in Olsztyn, 10-719 Olsztyn, Poland; natalia.kasica@uwm.edu.pl (N.K.)
3 Independent Laboratory of Behavioral Studies, Medical University of Lublin, 20-093 Lublin, Poland; monika.maciag@umlub.pl (M.M); barbara.budzynska@umlub.pl (B.B)
4. Biothermodynamics, TUM School of Life Sciences, Technical University of Munich, 85354 Freising, Germany; vlad.luca@tum.de (S.V.L)
5 Department of Pharmacognosy, *Grigore T. Popa* University of Medicine and Pharmacy Iasi, 700115 Iasi, Romania
6 Department of Pharmacy, Faculty of Medicine, University of Prishtina, 10000 Prishtina, Kosovo; Dafina.fondaj1@com (D.F.)
7 Department of Pathophysiology, Forensic Veterinary Medicine and Administration, Faculty of Veterinary Medicine, University of Warmia and Mazury in Olsztyn, 10-719 Olsztyn , Poland; piotr.podlasz@uwm.edu.pl (P.P)
8 Department of Chemistry of Natural Products, Medical University of Lublin, 20-093 Lublin, Poland; kskalicka@pharmacognosy.org (K.S.-W.)

* Correspondence: jwidelski@pharmacognosy.org (J.W.); kskalicka@pharmacognosy.org (K.S.W.)

Citation: Widelski, J.; Kasica, N.; Maciąg, M.; Luca, S.V.; Budzyńska, B.; Fondai, D.; Podlasz, P.; Skalicka-Woźniak, K. Simple Coumarins from *Peucedanum luxurians* Fruits: Evaluation of Anxiolytic Activity and Influence on Gene Expression Related to Anxiety in Zebrafish Model. *Int. J. Mol. Sci.* **2023**, *24*, 8693. https://doi.org/10.3390/ijms24108693

Academic Editors: Marta Anna Szychlinska and Antonella Marino Gammazza

Received: 21 March 2023
Revised: 30 April 2023
Accepted: 8 May 2023
Published: 12 May 2023

Copyright: © 2023 by the authors. Licensee MDPI, Basel, Switzerland. This article is an open access article distributed under the terms and conditions of the Creative Commons Attribution (CC BY) license (https://creativecommons.org/licenses/by/4.0/).

The influence of coumarins isolated from *Peucedanum luxurians* on spontaneous locomotor activity and thigmotaxis behavior of the zebrafish larvae.

The influence of officinalin on spontaneous locomotor activity and thigmotaxis behavior of the zebrafish larvae during 40 min light phase.

The spontaneous locomotor activity was measured as the average distance (in mm) moved during 40 minutes of continuous illumination. One-way ANOVA ($F (7, 236) = 5.599$; $p=0.0002$) revealed that officinalin at the concentration 6.0 μM decreased spontaneous locomotor activity **(Figure S1a)**.

Furthermore, one-way ANOVA showed statistically significant differences between the tested group when the distance moved in the central arena during 40 minutes of continuous light was considered ($F (7, 236) = 3.872$; $p=0.0041$) **(Figure S1b**). Post-hoc Tuckey's test revealed that diazepam increased the percentage of distance moved ($p<0.05$) in central arena in comparison to control group (DMSO-treated group) (**Figure S1b**).

One-way ANOVA showed also statistically significant differences in time spent in the central arena [$F (7, 236) = 1.931$; p=0,0981] in comparison to the DMSO-treated group (control group) (**Figure S1c**). Post-hoc Tuckey's test indicated that diazepam increased the percentage of time spent ($p<0.05$) in central arena (**Figure S1c).**

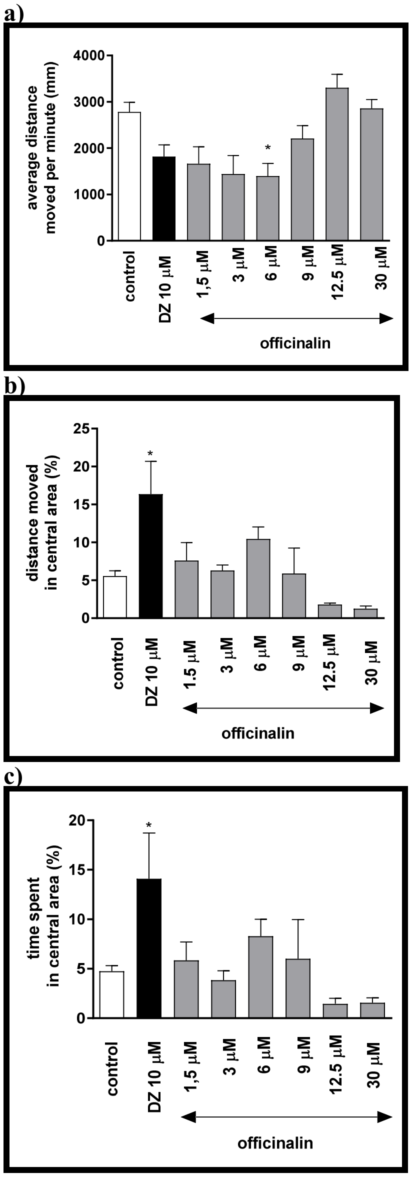

Figure S1. The effect of officinalin (1.5, 3, 6, 9, 12.5, 30 µM) and diazepam (DZ 10 µM) on locomotor activity during 40 min light phase. (**a**) Average distance (mm) moved by zebrafish larvae within each 1-min time bin; (**b**) the percentage of the distance moved in the central arena under continuous illumination; (**c**) the percentage of the time spent in the central arena under the continuous illumination. Data are presented as mean ± SEM, n=30, *p<0.01 in comparison to the control group, post hoc Tukey's test.

The influence of stenocarpin isobutyrate on spontaneous locomotor activity and thigmotaxis behavior of the zebrafish larvae during 40 min light phase.

The spontaneous locomotor activity was measured as the average distance (in mm) moved during 40 minutes of continuous illumination. One-way ANOVA (F (7, 236) = 0,5513; p=0.7894) revealed that stenocarpin isobutyrate in all concentrations (1.5, 3.0, 6.0, 9.0, 12.5 and 30 µM) has not influenced the spontaneous locomotor activity **(Figure S2a)**.

Furthermore, one-way ANOVA showed statistically significant differences between the tested group when the distance moved in the central arena during 40 minutes of continuous light was considered (F (7, 236) = 1.430; p=0.2258) **(Figure S2b**). Post-hoc test (Tukey's test) confirmed that diazepam increased (p<0.05) the percentage of distance moved in central arena in comparison to DMSO-treated group (**Figure S2b).**

One-way ANOVA showed also statistically significant differences in time spent in the central arena [F (7, 236) = 7,449; p<0.0001] in comparison to DMSO-treated group (control group) (**Figure S2c**). Post-hoc test (Tukey's test) confirmed that diazepam increased (p<0.01) the percentage of time spent in central arena (**Figure S2c).** Moreover, post-hoc Tukey's test showed that stenocarpin isobutyrate decreased the percentage of time spent in central arena in dose 6.0,9.0, 12.5 (p<0.05) and 30 µM (p<0.01) in comparison to control group (DMSO-treated group) (**Figure S2c**).

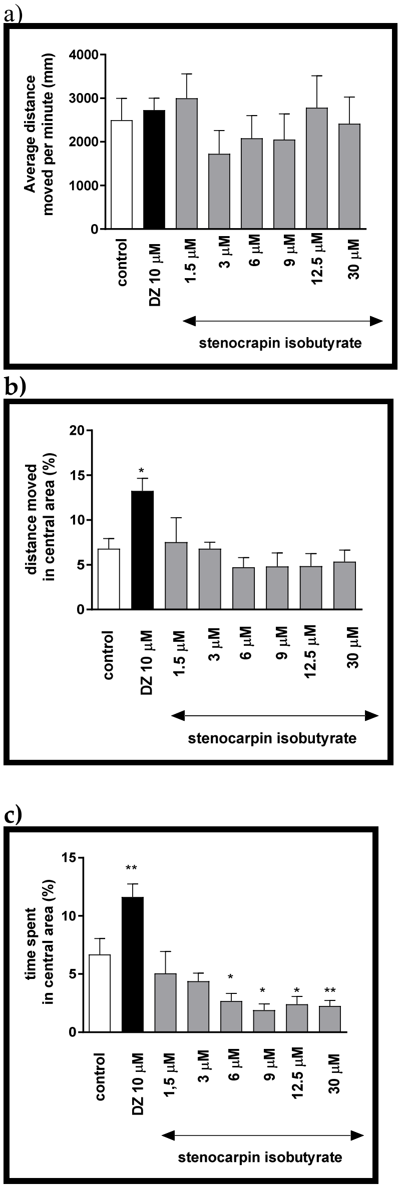

Figure S2. The effect of stenocarpin isobutyrate (1.5, 3, 6, 9, 12.5, 30 µM) and diazepam (DZ 10 µM) on locomotor activity during 40 min light phase. (**a**) Average distance (mm) moved by zebrafish larvae within each 1-min time bin; (**b**) the percentage of the distance moved in the

central arena under continuous illumination; (**c**) the percentage of the time spent in the central arena under the continuous illumination. Data are presented as mean ± SEM, n=30, *p<0.05, **p<0.01 in comparison to the control group, post hoc Tukey's test.

The influence of officinalin isobutyrate on spontaneous locomotor activity and thigmotaxis behavior of the zebrafish larvae during 40 min light phase.

The spontaneous locomotor activity was measured as the average distance (in mm) moved during 40 minutes of continuous illumination. One-way ANOVA [F (7, 250) = 0.9745, p=0.4645 revealed that stenocarpin isobutyrate in all concentrations (1.5, 3.0, 6.0, 9.0, 12.5 and 30 μM) has not influenced the spontaneous locomotor activity **(Figure S3a)**.
Furthermore, one-way ANOVA showed statistically significant differences between the tested group when the distance moved in the central arena during 40 minutes of continuous light was considered (F (7, 250) = 4.559, p=0.011) **(Figure S3b**). Post-hoc test (Tukey's test) revealed that diazepam increased (p<0.05) the percentage of distance moved in central arena in comparison to DMSO-treated group (**Figure S3b).**

One-way ANOVA showed also statistically significant differences in time spent in the central arena [F (7, 250) = 6.439, p=<0.0001] in comparison to DMSO-treated group (control group) (**Figure S3c**). Post-hoc test (Tukey's test) indicated that diazepam increased (p<0.05) the percentage of time spent in central arena in comparison to DMSO-treated group (**Figure S3c).** Moreover, post-hoc Tuckey's test showed that officinalin isobutyrate at concentration 30 μM decreased (p<0.05) time spent in central arena in comparison to DMSO-treated (control) group (**Figrue S3c)**

a)

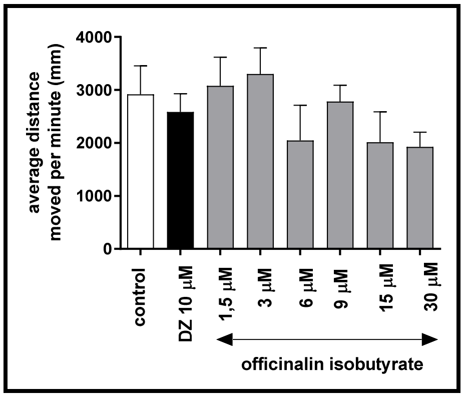

b)

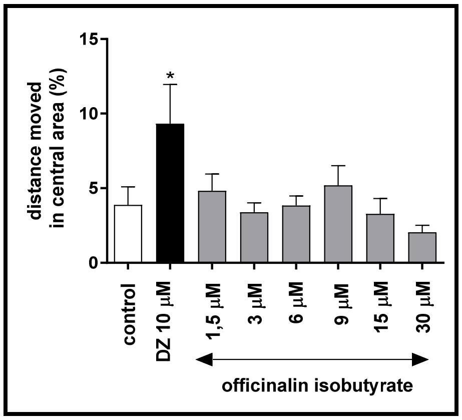

c)

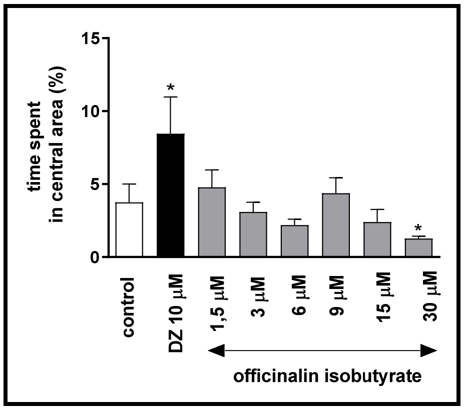

Figure S3. The effect of officinalin isobutyrate (1.5, 3, 6, 9, 12.5, 30 µM) and diazepam (10 µM) on locomotor activity during 40 min light phase. (**a**) Average distance (mm) moved by zebrafish larvae within each 1-min time bin; (**b**) the percentage of the distance moved in the central arena under continuous illumination; (**c**) the percentage of the time spent in the central arena under the continuous illumination. Data are presented as mean ± SEM, n=32, **$p<0.01$ in comparison to the control group, post hoc Tukey's test.
